# Large Language Models in Infectious Diseases: A Systemic Review

**DOI:** 10.21203/rs.3.rs-8901882/v1

**Published:** 2026-02-18

**Authors:** Alon Gorenshtein, Eyal Klang, Jacob J. Smith, Richard Dzeng, Mark C. Poznansky, Girish N Nadkarni, Mahmud Omar

**Affiliations:** Department of Neurology, Harvard Medical School, Boston, MA; The Windreich Department of Artificial Intelligence and Human Health, Mount Sinai Medical Center, NY, USA.; Vaccine and Immunotherapy Center, Massachusetts General Hospital, Boston, MA 02129, USA; Vaccine and Immunotherapy Center, Massachusetts General Hospital, Boston, MA 02129, USA; Vaccine and Immunotherapy Center, Massachusetts General Hospital, Boston, MA 02129, USA; The Windreich Department of Artificial Intelligence and Human Health, Mount Sinai Medical Center, NY, USA.; The Windreich Department of Artificial Intelligence and Human Health, Mount Sinai Medical Center, NY, USA.

**Keywords:** Infectious diseases, Large language models, Clinical decision support, Antimicrobial stewardship, Hallucinations (AI), Patient safety, Retrieval-augmented generation, Bias and fairness

## Abstract

**Background:**

Clinical reasoning in infectious diseases relies on validated evidence. LLMs are being introduced into diagnosis, antimicrobial stewardship, and guideline interpretation before their safety and reliability are established.

**Methods:**

This review, registered in PROSPERO (CRD420251155354), evaluated studies using GPT, Claude, Gemini, and retrieval-augmented or agentic systems for infectious disease decision-making. PubMed, CENTRAL, Scopus, and Web of Science were searched from January 2018 to September 2025. Two reviewers screened and extracted data. Risk of bias was assessed with QUADAS-AI.

**Findings:**

Thirty-one studies met inclusion criteria. Most were cross-sectional (61%) and vignette-based (68%). Only 32% used real clinical data; 23% had low risk of bias. Safety issues were reported in 90% of studies: incomplete responses (61%), unsafe advice (23–32%), and fabricated content (32%). In antimicrobial stewardship, agreement with infectious-disease specialists was ~ 50%. Diagnostic sensitivity for structured infections was 80–100%. Retrieval-augmented systems increased specificity from 35% to 75% and reduced hallucinations. Proprietary models outperformed open-source models but did not reach expert accuracy.

**Interpretation:**

LLMs perform well in defined diagnostic tasks but remain unreliable for autonomous clinical use. High error rates, inconsistent reasoning, and fabricated content require expert oversight and external validation before deployment.

## Main Point Summary

Large language models show promise in infectious diseases for narrow diagnostic tasks, surveillance, and stewardship, but most evidence is retrospective and high risk of bias. Hallucinations and context errors persist. External validation, human oversight, and retrieval-augmented or agentic safeguards are essential.

## Introduction

Clinical reasoning in infectious diseases (ID) depends on data, expertise, and judgment. Large language models (LLMs) now attempt to perform these tasks in text, generating diagnostic and therapeutic suggestions without task-specific training.^1–3^

ID present a natural testing ground. The specialty combines complex decision-making, workforce shortages, and rising antimicrobial resistance (AMR).^4,5^ The clinical burden is high across settings, with infections accounting for roughly 10% of ambulatory encounters in the USA (higher in paediatric care) and healthcare-associated infections affecting about 3% of hospitalized patients in point-prevalence surveys, yet specialist access is uneven, as over 80% of counties lack an ID physician and many fellowship positions go unfilled.^6–8^ Against this backdrop global AMR caused an estimated 4.95 million deaths in 2019, concentrated in respiratory and bloodstream infections.^9^

This environment invites automation. LLMs have been proposed for surveillance, guideline interpretation, and epidemic prediction.^10–15^ Yet early reports show instability—hallucinations, unsafe recommendations, and vulnerability to manipulation.^16–18^

This review examines whether recent advances have turned LLMs into reliable instruments for ID care.

## Methods

### Protocol and reporting

This systematic review was pre-registered with PROSPERO (CRD420251155354) and conducted according to PRISMA-2020 guidelines.^1^ Although quantitative pooling was pre-specified in the protocol, substantial heterogeneity in populations, interventions, and outcomes precluded meta-analysis.

### Eligibility criteria

We included primary research studies evaluating LLMs-including general-purpose models (e.g. ChatGPT, Gemini etc.), fine-tuned LLMs, retrieval-augmented generation (RAG) systems, and agentic LLMs-applied to ID decision-making in human clinical or public-health contexts.

Eligible study designs were original research articles. Review papers, case reports, conference abstracts, editorials, preprints, and studies not conducted in English were excluded. Additionally, we excluded studies of classic machine learning or natural language processing methods without an LLM component, image-only models without LLM reasoning, non-ID applications, purely administrative or educational uses, and synthetic cases not traceable to real clinical scenarios. Studies focusing solely on user satisfaction without clinical correctness were also excluded.

### Information sources and search

We searched PubMed, CENTRAL, Scopus, and Web of Science from 1 January 2018 to 30 September 2025. The search strategy combined index terms and free-text words for: (1) types of healthcare-associated infection and causative organisms; (2) LLM-based interventions, including IPC Core Components; (3) national or subnational context; and (4) outcome measures. No language restrictions were applied. Exact search strategies are provided in the appendix.

### Study selection

Two independent reviewers screened titles (A.G., M.O.), abstracts, and full texts using pre-defined eligibility criteria. Disagreements were resolved by discussion or consultation with a third reviewer (E.K.).

### Data extraction

Two reviewers (A.G. and M.O.) independently extracted key study details, including design, task type, disease focus, model used, data source, validation, comparator, and main outcomes. Unclear or missing information was clarified with authors or recorded as “not reported.”

### Risk of bias and reporting quality

We assessed risk of bias using QUADAS-AI.^2^ Two reviewers (A.G.,M.O.) independently assessed each domain; disagreements were resolved by discussion.

### Data synthesis

We synthesized findings narratively, stratified by task category, disease focus, and LLM family. Within task categories, we grouped studies by specific infections (e.g., pneumonia/respiratory, bloodstream infection [BSI]/sepsis, catheter-associated urinary tract infection [CAUTI], invasive fungal infection [IFI/IMD], HIV/sexually transmitted infections [STIs], tuberculosis [TB]). When three or more commensurable studies examined the same task, disease, and metric, we reported descriptive statistics (median [IQR]) without formal meta-analytic pooling.

## Results

### Study selection

**Figure 1:**
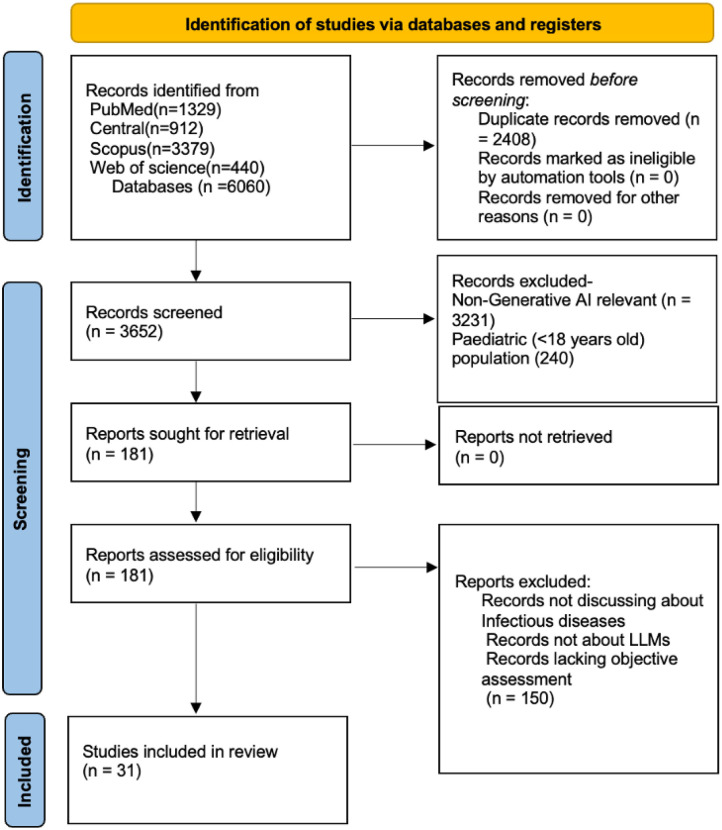
PRISMA Flow Diagram

The search retrieved 6,060 records from PubMed (1,329), CENTRAL (912), Scopus (3,379), and Web of Science (440). After removing 2,408 duplicates, 3,652 records were screened. Of these, 3,471 were excluded for irrelevance or pediatric focus. The remaining 181 full texts were reviewed, and 150 were excluded for not meeting inclusion criteria. Thirty-one studies, published between 2023 and 2025, were included in the final analysis (Figure 1).^3–33^

### Risk of bias

QUADAS-AI assessment revealed low overall risk in 7/31 studies (23%) and high risk in 24/31 (77%) (**Table Sup 1**). Patient selection showed high risk in 24 studies (77%), primarily attributable to reliance on synthetic vignettes or curated guideline-based questions rather than consecutive real patient encounters. Meanwhile, the remaining domains exhibited predominantly low risk: index test (24/31 low risk, 77%), reference standard (27/31 low risk, 87%), and flow and timing (29/31 low risk, 94%).

### Study characteristics

The 31 included studies evaluated LLM applications across diverse settings: inpatient wards (n=8), ICU (n=1), outpatient or primary care (n=1), mixed clinical settings (n=10), public health (n=2), laboratory (n=1), and comparative bench/question-based studies (n=8). Study designs comprised cross-sectional evaluations (n=19), prospective studies (n=5), retrospective analyses (n=4), and specialized accuracy/pilot studies (n=3).

Disease foci included sexually transmitted diseases (n=4), urinary tract infections (n=4), tuberculosis (n=2), sepsis (n=2), HIV (n=2), hepatitis (n=2), mixed ID (n=3), and single studies on bacteremia, osteoarticular infections, vertebral osteomyelitis, blood culture diagnosis, surgical site infection, pneumonia, antimicrobial resistance, Infection Prevention and Control(IPC) guidelines, infective endocarditis prophylaxis, and general ID topics. Sample sizes ranged from 6 vignettes to 4,786 patients.

LLM families included GPT-4/4o (n=20 studies), Claude 3/3.5 (n=7), Gemini (n=10), Mistral/Llama (n=3), custom chatbots (n=2), and specialized tools (n=6 studies using Consensus, OpenEvidence, Amboss, Perplexity, Microsoft Copilot). Retrieval-augmented generation or agentic architectures were explicitly implemented in 5 studies.

Data sources were vignettes/synthetic cases (n=21) and real EHR or clinical data (n=10). Comparators included infectious disease specialists (n=8), guidelines (n=8), other LLMs (n=13), or none (n=2) (**Supplementary Table 2**).

### Safety and Clinical Applicability

Safety concerns were documented in 28/31 studies (90.3%). Clinically incomplete responses represented the most prevalent issue (19/31, 61.3%), with usefulness ratings of 42% and mean completeness scores of 40%. Unsafe recommendations occurred in 10/31 studies (32.3%), manifesting as substandard responses posing serious health risk in 10.3% of evaluated cases. Harmful or inadequate recommendations appeared in 7/31 studies (22.6%): 16% potentially hazardous bacteremia management plans (Maillard et al.)^32^, 71% incorrect isolation precautions in sepsis cases (Lorenzoni et al.)^3^, and 9% inadequate source-control recommendations rates significantly exceeding infectious disease expert benchmarks (1–4%, p<0.05).^32^

Hallucinations (fluent, confident response that presents incorrect information, akin to clinical confabulation), fabricated citations, contradictory statements, or artificial clinical details were documented in 10/31 studies (32.3%). Constraint-induced limitations (guardrail refusals, unanswered queries, token limits preventing processing of complete medical records) affected 9/31 studies (29%): 8% of interactions yielded no response and 65% experienced extraction errors from incomplete chart access. Context-dependent failures (errors mitigated by providing complete clinical data or external knowledge bases) in 3 studies, (3/31, 9.7%) demonstrated marked improvement with interventions: retrieval-augmented generation reduced hallucinations; providing complete clinical documentation improved central line-associated bloodstream infection detection specificity from 35% to 75% (Rodriguez-Nava et al.). These findings indicate requirements for expert oversight, structured validation protocols, and context-enrichment architectures before clinical deployment (Supplementary Table 2).

### Antimicrobial Stewardship Performance

Twelve studies assessed LLMs in antimicrobial stewardship. Across tasks, concordance with infectious-disease specialists was moderate, averaging about 50%. Two bacteremia vignette studies (n=100 each) reported identical 51% agreement (κ=0.48). Agreement was higher for Gram-positive (70%, κ=0.68) than Gram-negative infections (46%, κ=0.43). In Maillard et al.’s prospective study using real patient data (n=44, GPT-4), diagnostic accuracy reached 59% and empiric therapy appropriateness 64%, but 16% of recommendations were potentially harmful, including inactive agents and missed source-control interventions.^32^ In a blood culture stewardship study (n = 84), LLMs produced 13% harmful or inadequate recommendations, significantly higher than experts (4%, p = 0.047). Most errors involved missing echocardiography for suspected endovascular infections.^24^

A 14-model comparison showed wide variation in antibiotic prescribing accuracy. Antibiotic selection ranged from 30% to 100%, while dose and duration accuracy fell to 0–92%. Citation accuracy ranged 0–100%, and several models produced fabricated references. ChatGPT-o1 performed best overall (71.7% correct, 43/60).^30^ In real-patient testing, Lorenzoni et al. (n = 7, GPT-4o) achieved perfect concordance for antibiotic selection but misjudged isolation precautions in 71% of cases.^3^ Rodriguez-Nava and colleagues reported initially low central line-associated bloodstream infection (CLABSI) detection specificity (35%), which improved to 75% when complete chart information was provided, highlighting the critical role of RAG in improving accuracy.^11^ In outpatient vignettes (n = 24, six models), Nguyen et al. found correct antibiotic selection between 59% and 100% and complete clinical advice between 25% and 96%, with proprietary models outperforming open-source ones.^29^

### Diagnostic Accuracy and Guideline Concordance

Four studies evaluated LLM diagnostic accuracy for infectious conditions using sensitivity and specificity metrics. Diagnostic sensitivity ranged from 80% to 100% (median 91%), with high performance for catheter-associated urinary tract infection detection (91%), tuberculous pleural effusion diagnosis (89%), and surgical site infection screening (100%). Specificity varied substantially (range 35–100%, median 92%). Wu and colleagues demonstrated that a custom LLM for tuberculous pleural effusion diagnosis achieved AUROC 0·96 (sensitivity 76%, specificity 100%), matching or exceeding traditional machine learning models.^17^ Satheakeerthy and colleagues showed zero-shot Llama-3–70B could screen surgical site infections with 100% sensitivity and 86% specificity, flagging infections earlier than infection control staff in 50% of cases.^12^

**Figure 2:**
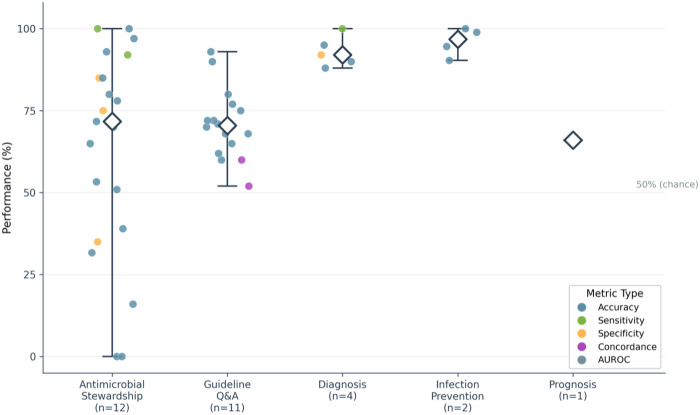
Performance of large language models across clinical task categories in infectious diseases: Performance metrics reported across 30 studies evaluating large language models in infectious diseases, stratified by clinical task category. Each point represents an individual performance metric from a single study, with colour indicating metric type: accuracy (blue), sensitivity (green), specificity (orange), concordance (purple), or AUROC (grey). White diamonds indicate median performance within each task category. Vertical lines represent the range from minimum to maximum reported values. Numbers in parentheses denote the number of studies contributing to each category. Performance varied substantially within and across task categories, with antimicrobial stewardship and guideline-based question-answering showing the widest ranges (0–100% and 21–93%, respectively), while diagnosis and infection prevention tasks demonstrated more consistently high performance (medians >90%). The single prognosis study reported AUROC of 66% for sepsis mortality prediction, marginally above chance level (50%, indicated).

Twelve studies evaluated LLMs in answering guideline-based questions, with accuracy ranging from 42% to 98% depending on topic and complexity. Borgonovo et al. found specialized RAG tools (Open Evidence, Microsoft Copilot) most accurate (94.4%), outperforming general-purpose models such as GPT-4o and Gemini 2.5 Pro (92.9%).^26^ Lin et al. showed OpenAI O1 performed best for pneumonia guidelines, achieving 55% “excellent” responses and effective self-correction, compared with GPT-4o, which produced 40% “poor” responses.^20^ Kufel et al. reported only 41.8% of GPT-3.5 outputs were rated “useful,” with low completeness (5.8/10) and safety (6.4/10).^28^ Accuracy also varied by disease: tuberculosis questions scored 3.6–4.4/5, viral hepatitis 71–78%, and infective endocarditis prophylaxis 69–80%, with GPT-4o highest (80%). Performance was consistently better for informal, social-media-style questions (92%) than for formal guideline queries (69%, p < 0.001) (Figure 2, **Supplementary Table 3.**).^9^

### Limitations and Methodological Quality

Methodological quality was constrained by design and reporting deficiencies. More than two-thirds of studies (21/31, 68%) evaluated LLMs using synthetic vignettes or curated cases, potentially overestimating performance by avoiding complexity, ambiguity, and incomplete documentation characteristic of actual practice. Among studies using real patient data (32%), most were retrospective with attendant selection bias. Sample sizes were frequently inadequate (35%), yielding unstable estimates. Validation approaches were weak across the evidence base. 12 studies (39%) had no expert comparison, instead benchmarking LLMs against other LLMs or guideline text alone. 14 studies (45%) judged correctness by guideline concordance without clinical context. The predominant cross-sectional design (22/31, 71%) precluded assessment of performance stability over time, while lack of blinding in 24/31 (77%) introduced expectation bias that might favor novel technology. Reproducibility and transparency were deficient. Most studies queried each question only once without assessing response variability, potentially concealing inconsistency and raising concerns about selective reporting of optimal responses. Process measures were absent in 21/31 (68%), leaving intervention fidelity and implementation strategies unclear. Outcome definitions were universally heterogeneous, employing non-standardized custom scales for usefulness, appropriateness, or quality that precluded cross-study comparison or meta-analysis (Table 2).

## Discussion

In this systematic review of 31 studies, we found wide variability in how LLMs perform across infectious disease medical practice. Their accuracy and reliability remain insufficient for autonomous clinical use. Across antimicrobial stewardship, diagnosis, guideline interpretation, and surveillance tasks, performance was inconsistent. Concordance with infectious-disease specialists for empiric therapy averaged about Taken together, the evidence suggests that LLM diagnostic performance is strongest for narrow, well-specified classification tasks, ranging from common syndromes such as CAUTI,SSI to rarer entities such as tuberculous pleural effusions. However, accuracy and calibration consistently decline when models must integrate context across comorbidities, timelines, devices, cultures, and competing diagnoses, which is where clinical reasoning is most vulnerable to error. According to current evidence, RAG can improve specificity by providing access to full clinical documentation. Yet safety remains a central limitation: most studies reported incomplete or unsafe recommendations and, in many cases, fabricated or contradictory content. Even the most advanced models (at time of evaluation), such as GPT-4o and Claude 3.5, performed better than open-source systems but still failed to reach expert reliability. Their stronger results on conversational or social-media-style questions compared with formal guideline queries suggest training data skewed toward lay information rather than specialist clinical knowledge.

### Safety and clinical applicability

**Figure 3:**
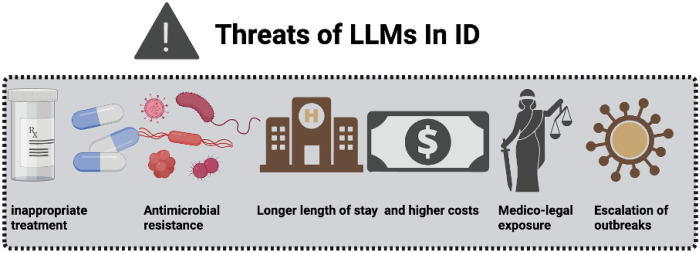
Threats of Large Language Models in Infectious Diseases: Inaccurate or context-blind outputs can drive inappropriate treatment, antimicrobial resistance, longer hospital stays and costs, medico-legal exposure, and outbreak escalation, showcasing the need for expert oversight, validated RAG, and regulation before clinical deployment.

Safety has emerged as a significant barrier to the clinical deployment of LLMs in ID management. Rates of harmful or inadequate antimicrobial recommendations from these models ranged between 13% and 16%, sharply contrasting with the 1% to 4% error rates reported by infectious disease experts (Maillard et al.). Such harmful recommendations were described in Schwartz et al. document a paradigm case: when prompted to create a management plan for cryptococcal meningitis, GPT-3.5 recommended initiating antiretroviral therapy within 2 weeks-a recommendation^34^ directly contradicted by Boulware et al.’s randomized controlled trial proving this approach increases mortality.^35^ Such inaccuracies pose serious risks by jeopardizing patient safety and exacerbating the global issue of AMR.^36^ The underlying causes of these errors may mirror those observed in AMR challenges globally, primarily arising from various factors, such as wrong indication, selection, dosage, duration, lack of adherence to infection prevention and control (IPC) protocols.^37,38^ LLMs showed this issues when they were lacking a defined guidelines, leading to generate incorrect responses. This challenge is further compounded by geographic variability in treatment protocols (Nguyen et al.); as established LLMs often lack access to localized medical knowledge.^39^ This highlights the imperative for implementing RAG systems, which would enable LLMs to integrate context-specific information, thereby aligning with established protocols and mitigating the risk of adverse treatments. Another contributing factor to these inaccuracies is the inherent issue of hallucinations generated by LLMs.^40^ While not errors per se (byproduct of LLMs training)^41^, these hallucinations manifesting as spurious guideline citations, contradictory assertions within a single interaction, or fabricated clinical details were documented in approximately one-third of the studies reviewed, undermining clinical trust and introducing medico-legal risks. This phenomenon is linked to LLMs’ limited access to real-time resources.^42^ However, research indicates that providing web access significantly enhances their ability to generate accurate, high-quality scientific references.^43^ Given these considerations, LLMs should not be deployed in their unmodified form due to their potential threats in the field (Figure 3.). Instead, they should be utilized as part of AI agents that leverage LLM capabilities while planning tasks, accessing external tools, and coordinating with other agents. In contrast to standard LLMs, these agents can perform multi-step processes, access real-time clinical information, and synthesize data from diverse sources.^44^ This approach addresses the aforementioned safety concerns while also tackling additional issues such as verbosity, the need for expert-in-the-loop safety mechanisms, and iterative improvement. Due to the dearth of studies focusing specifically on ID, we cannot assertively conclude that such AI agents will resolve these challenges. However, this represents a critical area for future research as the next phase of LLM studies in infectious disease should aim to explore these innovations.

CAUTI and CLABSI exemplify both the promise and the ceiling for LLM-enabled hospital epidemiology. These endpoints are operational quality metrics defined by NHSN surveillance rules,^45,46^ not bedside diagnoses, so adjudication hinges on consistent application of standardized criteria to temporally ordered device, culture, and symptom data, often under substantial infection-prevention workload.^21^ In CAUTI, GPT-4 achieved high performance on curated case abstractions and improved further with iterative, criteria-aligned prompting, highlighting how structured inputs and explicit rule framing can materially shift reliability. In contrast, CLABSI identification from real clinical notes was strongly context dependent: when the model was constrained to partial chart excerpts, sensitivity remained high but specificity was limited, and performance improved when key missing chart elements were supplied, supporting the use of RAG to pull the relevant EHR fields and NHSN rule elements before generation. Near term, LLMs are best deployed as definition-aware tools for education and structured abstraction that priorities sensitivity and trigger escalation, while final CAUTI and CLABSI attribution remains with trained infection-prevention reviewers. Because surveillance labels can propagate to antimicrobial decisions, CAUTI workflows should include explicit stewardship safeguards that confirm symptoms and exclude asymptomatic bacteriuria before outputs are acted on.

### Comparison with prior literature

Up until now the topic of AI in ID is residing in a controversial place. A stark example is the contradiction between Siddig et al. declaring AI “revolutionizing” ID control^47^ versus Schwartz’s et al. “Black Box Warning” arguing “existing LLMs are not safe for clinical consultation”.^34^

Our own group’s previous systematic review of 15 studies identified promise of NLP and LLM in areas like pathogen detection and surveillance but noted limited real-world validation.^48^ Our current findings extend beyond that review which included by the time of publications only two LLM studies. Be it as it may our current results show persistence of LLM limitation in literature, as evidenced by our observation that 58% of included studies relied on synthetic vignettes rather than real-world data.

Howard et al. highlighted similar challenges in data completeness and interoperability for AI in tackling AMR, advocating for support of UN General Assembly targets like antimicrobial stewardship programs and surveillance, though with less focus on LLM-specific safety risks.^49^ Despite this, our review found that LLMs may be useful in specific niches: diagnostics for urinary tract infections (UTIs), pneumonia, bloodstream infections (BSIs), and invasive fungal infections in defined populations (median AUROC 0.82, range 0.64–0.95); social media-based disease surveillance (accuracy 85–100%, with 92% for informal queries); antimicrobial stewardship (median appropriateness 71%, range 57–85%); and infection prevention/control with structured prompts (accuracy 98–100%). These successes share common features of well-defined diagnostic criteria, structured data sources, supplementary human verification, and consequences of errors that allow correction before patient harm.

### Limitations

Our systematic review has several limitations. First, substantial heterogeneity in populations, interventions, and outcome measures precluded meta-analysis. Second, the overall risk of bias was high across included studies. Third, the rapidly evolving nature of LLM technology means newer models might show different performance characteristics than those evaluated in our included studies. Finally, the predominance of retrospective studies in our review indicates a need for more prospective studies to validate the findings.

## Conclusions

The reviewed iterations of LLMs are not well-suited for clinical application in ID. Most studies highlight safety concerns despite the models demonstrating high performance in structured tasks (UTI, BSI and fungal infections). Issues such as hallucinations, missing guideline information, and lack of web search capabilities contribute to misinformation from LLMs. To mitigate these challenges, LLMs should be employed as AI agents before being utilized in future studies.

## Supplementary Material

Supplementary Files

This is a list of supplementary files associated with this preprint. Click to download.

- FullAPPENDIXIDFinalFeb.pdf

## Figures and Tables

**Table 1.** Safety issue categories, prevalence across studies, quantitative signals, and mitigations

Safety issue category	Studies (n/31; %)	Representative quantitative rate(s)	Severity tier	Context-dependent?	Clinical risk domain(s)	Common mitigations	Example studies
Unsafe recommendations	10/31 (32.3%)	7/68 (10.3%) substandard responses posing serious health risk	High	Yes	Guideline adherence, dosing, antimicrobial choice, AMR interpretation	Expert review, external validation	Abosi 2024, Cakir 2023
Harmful/inadequate recommendations	7/31 (22.6%)	7/44 (15.9%) harmful management plans; 5/7 (71.4%) incorrect isolation precautions; 4/44 (9.1%) inadequate source control	High	Yes	Isolation/IPC, antimicrobial choice, dosing, source control	Prompt engineering, chain-of-thought prompting, expert review	Lorenzoni 2025, Maillard 2024
Hallucinations / fabricated citations	10/31 (32.3%)	not reported	Moderate	Yes	Antimicrobial choice, dosing, citations	RAG to guidelines, external knowledge bases	Borgonovo 2025, Cakir 2023
**Inadequate or incomplete responses**							
Clinically incomplete	19/31 (61.3%)	209/500 (41.8%) responses deemed not useful; 8/20 (40.0%) poor guideline adherence; 42/100 (42.0%) AMR mechanism errors	Moderate	Yes	Isolation/IPC, guideline adherence, antimicrobial choice, citations, AMR interpretation, diagnostics, dosing	Chain-of-thought prompting, prompt engineering, external validation	Abosi 2024, Kufel 2024
Constraint-induced	9/31 (29.0%)	31/393 (7.9%) unanswered queries; 11/17 (64.7%) errors from missing chart information	Low	Yes	Diagnostics, extraction	RAG, expanded context windows, structured input templates	Lorenzoni 2025, Rodriguez-Nava 2025
Internal contradictions / instability	2/31 (6.5%)	not reported	Moderate	Unclear	Guideline adherence, antimicrobial choice	Version pinning, multiple runs with consensus	Cakir 2023, Borgonovo 2025
Context-dependent failures	3/31 (9.7%)	Specificity improved from 35% to 75% when complete chart provided	Moderate	Yes	Diagnostics, surveillance	RAG, full chart access, external data integration	Rodriguez-Nava 2025, Wiemken 2024
Any safety event	**28/31 (90.3%)**	—	—	—	—	—	—

1. **Definitions:** Unsafe recommendations = clearly incorrect/contraindicated management; Harmful/inadequate = omissions (e.g., source control) or dosing errors with potential for patient harm; Hallucinations = fabricated citations, contradictory statements, or artificial clinical details; Clinically incomplete = missing key clinical elements despite being prompted; Constraint-induced = refusals, guardrail blocks, verbosity, token limits, or language barriers preventing complete responses; Internal contradictions = intra-session inconsistencies in recommendations; Context-dependent = errors mitigated by providing full chart access or external knowledge.

2. Percentages reflect unique studies per category. Studies may appear in multiple categories if they reported multiple safety issue types.

3. “Not reported” indicates no numeric denominator was available in the source studies despite qualitative safety signals being documented.

4. “Context-dependent” indicates issues that improved with RAG, full chart access, or expert oversight in reported studies.

5. Common mitigations listed represent strategies that were tested and showed some benefit across studies, though not all were formally quantified.

**Table 2.** Study Limitations and Clinical Applicability Constraints

Limitation Category	Studies Affected (n/31; %)	Description and Examples	Impact on Clinical Applicability
**Study Design and Validation**			
Vignette/synthetic cases	21/31 (67.74%)	Simulated rather than real clinical scenarios; may not capture complexity	May overestimate performance in actual practice
Small sample size	11/31 (35%)	<50 cases evaluated (range: 7–44)	Insufficient power; wide confidence intervals
Single-center studies	8/31 (26%)	Limited to one institution’s practices/population	Generalizability uncertain across healthcare systems
**Data and Context Constraints**			
Language limitations	3/31 (10%)	Clinical documentation in non-English (Italian, Turkish); chatbots English-only	Reduced accuracy for non-English contexts; limits global applicability
Token/context limits	5/31 (16%)	Unable to process complete medical records; limited to recent 2 progress notes	Critical information missed; specificity dropped 35%→75% when full chart provided
Knowledge cutoff issues	4/31 (13%)	Training data ended 2021; inconsistent with latest guidelines (e.g., 2023 HBV consensus)	Outdated recommendations; missed recent evidence
Missing local context	6/31 (19%)	No integration of local antibiograms, formularies, or resistance patterns	Recommendations may be inappropriate/unavailable regionally
**Governance and Safety**			
No PHI protections	12/31 (39%)	Public web interfaces; no HIPAA-compliant/secure instances	Cannot be used with real patient data in most settings
IRB not required/waived	8/31 (26%)	Classified as non-human subjects research or quality improvement	Ethical oversight gaps for clinical deployment
Lack of regulatory alignment	23/31 (74%)	No FDA/CE marking or clinical validation pathway	Unclear regulatory status for clinical use
**Implementation and Usability**			
Verbosity/poor readability	5/31 (16%)	Responses 200–400+ words; reading level >10th grade vs recommended 6th grade	Unusable in time-critical settings; incomprehensible to patients
Inconsistent reproducibility	4/31 (13%)	Different answers when asked repeatedly; within-session contradictions	Unreliable for clinical decision-making
Constraint-induced failures	9/31 (29%)	Guardrail refusals, unanswered queries (8% of interactions), or excessive caveats	Incomplete clinical utility; frustrating user experience
No uncertainty quantification	6/31 (19%)	Failed to acknowledge limitations or express appropriate uncertainty	False confidence; safety risk
**Clinical Preparedness Gaps**			
No real-time EHR integration	27/31 (87%)	Manual data entry required; cannot access lab/imaging/notes automatically	Impractical for clinical workflow; introduces transcription errors
No human-in-the-loop validation	19/31 (61%)	No expert review mechanism before recommendations acted upon	Unsafe for autonomous use
Insufficient process measures	21/31 (68%)	Intervention fidelity not reported; unclear what drove observed effects	Cannot determine which implementation strategies effective

Percentages represent unique studies reporting each limitation category. Categories are not mutually exclusive. EHR=electronic health record; HBV=hepatitis B virus; IRB=institutional review board; PHI=protected health information.

## References

[1] Page MJ, McKenzie JE, Bossuyt PM (2021) The PRISMA 2020 statement: an updated guideline for reporting systematic reviews. BMJ 372:n71. 10.1136/bmj.n71 33782057 PMC8005924

[2] Guni A, Sounderajah V, Whiting P, Bossuyt P, Darzi A, Ashrafian H (2024) Revised Tool for the Quality Assessment of Diagnostic Accuracy Studies Using AI (QUADAS-AI): Protocol for a Qualitative Study. JMIR Res Protoc 13:e58202. 10.2196/58202 39293047 PMC11447435

[3] Lorenzoni G, Garbin A, Brigiari G, Papappicco CAM, Manfrin V, Gregori D (2025) Large Language Models in Action: Supporting Clinical Evaluation in an Infectious Disease Unit. Healthcare 13(8):879. 10.3390/healthcare13080879 40281830 PMC12027404

[4] Evaluation of artificial intelligence (AI) chatbots for providing sexual health information: a consensus study using real-world clinical queries | BMC Public Health | Full Text. Accessed October 8 (2025) https://bmcpublichealth.biomedcentral.com/articles/10.1186/s12889-025-22933-8 10.1186/s12889-025-22933-8 PMC12080182 40375254

[5] De Vito A, Geremia N, Marino A (2025) Assessing ChatGPT’s theoretical knowledge and prescriptive accuracy in bacterial infections: a comparative study with infectious diseases residents and specialists. Infection 53(3):873–881. 10.1007/s15010-024-02350-6 38995551 PMC12137519

[6] AI Chatbots as Sources of STD Information: A Study on Reliability and Readability | Journal of Medical Systems. Accessed October 8 (2025) https://link.springer.com/article/10.1007/s10916-025-02178-z 10.1007/s10916-025-02178-z PMC11968469 40178771

[7] Zhuo KY, Kim P, Kovacic J (2024) Can Artificial Intelligence Treat My Urinary Tract Infections?—Evaluation of Health Information Provided by OpenAI^™^ ChatGPT on Urinary Tract Infections. Société Int D’Urologie J 5(2):104–107. 10.3390/siuj5020018

[8] Cakir H, Caglar U, Sekkeli S (2024) Evaluating ChatGPT ability to answer urinary tract Infection-Related questions. Infect Dis Now 54(4):104884. 10.1016/j.idnow.2024.104884 38460761

[9] Rewthamrongsris P, Burapacheep J, Trachoo V, Porntaveetus T (2025) Accuracy of Large Language Models for Infective Endocarditis Prophylaxis in Dental Procedures. Int Dent J 75(1):206–212. 10.1016/j.identj.2024.09.033 39395898 PMC11806337

[10] Tunçer G, Güçlü KG (2024) How Reliable is ChatGPT as a Novel Consultant in Infectious Diseases and Clinical Microbiology? Infect Dis Clin Microbiol 6(1):55–59. 10.36519/idcm.2024.286 38633442 PMC11020004

[11] Rodriguez-Nava G, Egoryan G, Goodman KE, Morgan DJ, Salinas JL (2024) Performance of a large language model for identifying central line-associated bloodstream infections (CLABSI) using real clinical notes. Infect Control Hosp Epidemiol 46(3):1–4. 10.1017/ice.2024.164 PMC11883649 39473230

[12] Satheakeerthy S, Stretton B, Tsimiklis J Zero-shot large language model application for surgical site infection auditing. Infect Dis Health. Published online May 21, 2025:S2468–0451(25)00030 – 6. 10.1016/j.idh.2025.05.001 40404536

[13] Cheah MH, Gan YN, Altice FL (2024) Testing the Feasibility and Acceptability of Using an Artificial Intelligence Chatbot to Promote HIV Testing and Pre-Exposure Prophylaxis in Malaysia: Mixed Methods Study. JMIR Hum Factors 11:e52055. 10.2196/52055 38277206 PMC10858413

[14] Ni Z, Oh S, Saifi R, Azwa I, Altice FL (2025) Evaluating the Usability of an HIV Prevention Artificial Intelligence Chatbot in Malaysia: National Observational Study. JMIR Hum Factors 12:e70034. 10.2196/70034 40663792 PMC12283058

[15] Li Y, Huang CK, Hu Y, Zhou XD, He C, Zhong JW (2025) Exploring the performance of large language models on hepatitis B infection-related questions: A comparative study. World J Gastroenterol 31(3):101092. 10.3748/wjg.v31.i3.101092 39839898 PMC11684168

[16] Comparison of the performances between ChatGPT and Gemini in answering questions on viral hepatitis | Scientific Reports. Accessed October 8 (2025) https://www.nature.com/articles/s41598-024-83575-1 10.1038/s41598-024-83575-1 PMC11724965 39799203

[17] The large language model diagnoses tuberculous pleural effusion in pleural effusion patients through clinical feature landscapes | Respiratory Research | Full Text. Accessed October 8 (2025) https://respiratoryresearch.biomedcentral.com/articles/10.1186/s12931-025-03130-y 10.1186/s12931-025-03130-y PMC11823098 39939874

[18] Large (2025) language models’ capabilities in responding to tuberculosis medical questions: testing ChatGPT, Gemini, and Copilot | Scientific Reports. Accessed October 8. https://www.nature.com/articles/s41598-025-03074-9 10.1038/s41598-025-03074-9 PMC12102205 40410343

[19] Giske CG, Bressan M, Fiechter F (2024) GPT-4-based AI agents-the new expert system for detection of antimicrobial resistance mechanisms? J Clin Microbiol 62(11):e0068924. 10.1128/jcm.00689-24 39417635 PMC11559085

[20] Lin Z, Li Y, Wu M (2025) Performance analysis of large language models Chatgpt-4o, OpenAI O1, and OpenAI O3 mini in clinical treatment of pneumonia: a comparative study. Clin Exp Med 25(1):213. 10.1007/s10238-025-01743-7 40540092 PMC12181206

[21] Wiemken TL, Carrico RM (2024) Assisting the infection preventionist: Use of artificial intelligence for health care-associated infection surveillance. Am J Infect Control 52(6):625–629. 10.1016/j.ajic.2024.02.007 38483430

[22] Abosi OJ, Kobayashi T, Ross N (2024) A head-to-head comparison of the accuracy of commercially available large language models for infection prevention and control inquiries, 2024. Infect Control Hosp Epidemiol 46(3):1–3. 10.1017/ice.2024.205 PMC11883648 39664019

[23] Oh N, Cha WC, Seo JH (2024) ChatGPT Predicts In-Hospital All-Cause Mortality for Sepsis: In-Context Learning with the Korean Sepsis Alliance Database. Healthc Inf Res 30(3):266–276. 10.4258/hir.2024.30.3.266 PMC11333818 39160785

[24] Tassone DM, Hitchcock MM, Rossier CJ (2025) Evaluating chain-of-thought prompting in a GPT chatbot for BCID2 interpretation and stewardship: how does AI compare to human experts? Antimicrob Steward Healthc Epidemiol ASHE 5(1):e154. 10.1017/ash.2025.10059 40657035 PMC12247004

[25] Milicevic F, Ghandour M, Khasawneh MY (2025) Assessing LLMs on IDSA Practice Guidelines for the Diagnosis and Treatment of Native Vertebral Osteomyelitis: A Comparison Study. J Clin Med 14(14):4996. 10.3390/jcm14144996 40725688 PMC12295083

[26] Borgonovo F, Matsuo T, Petri F (2025) Battle of the Bots: Solving Clinical Cases in Osteoarticular Infections With Large Language Models. Mayo Clin Proc Digit Health 3(3):100230. 10.1016/j.mcpdig.2025.100230 40583928 PMC12205795

[27] Evaluating the Performance of State-of-the-Art Artificial Intelligence Chatbots Based on the WHO Global Guidelines for the Prevention of Surgical Site Infection: Cross-Sectional Study - PubMed. Accessed October 8 (2025) https://pubmed.ncbi.nlm.nih.gov/40744114/10.2196/75567 PMC12313333 40744114

[28] Kufel WD, Hanrahan KD, Seabury RW (2024) Let’s Have a Chat: How Well Does an Artificial Intelligence Chatbot Answer Clinical Infectious Diseases Pharmacotherapy Questions? Open Forum Infect Dis 11(11):ofae641. 10.1093/ofid/ofae641 39529938 PMC11551448

[29] Ngoc Nguyen O, Amin D, Bennett J (2025) GP or ChatGPT? Ability of large language models (LLMs) to support general practitioners when prescribing antibiotics. J Antimicrob Chemother 80(5):1324–1330. 10.1093/jac/dkaf077 40079276 PMC12046391

[30] De Vito A, Geremia N, Bavaro DF (2025) Comparing large language models for antibiotic prescribing in different clinical scenarios: which performs better? Clin Microbiol Infect Off Publ Eur Soc Clin Microbiol Infect Dis 31(8):1336–1342. 10.1016/j.cmi.2025.03.002 40113208

[31] Montiel-Romero S, Rajme-López S, Román-Montes CM (2025) Recommended antibiotic treatment agreement between infectious diseases specialists and ChatGPT^®^. BMC Infect Dis 25(1):38. 10.1186/s12879-024-10426-9 39773383 PMC11706082

[32] Maillard A, Micheli G, Lefevre L (2024) Can Chatbot Artificial Intelligence Replace Infectious Diseases Physicians in the Management of Bloodstream Infections? A Prospective Cohort Study. Clin Infect Dis Off Publ Infect Dis Soc Am 78(4):825–832. 10.1093/cid/ciad632 37823416

[33] Perret J, Schmid A (2024) Application of OpenAI GPT-4 for the retrospective detection of catheter-associated urinary tract infections in a fictitious and curated patient data set. Infect Control Hosp Epidemiol 45(1):96–99. 10.1017/ice.2023.189 37675518 PMC10782204

[34] Schwartz IS, Link KE, Daneshjou R, Cortés-Penfield N (2023) Black Box Warning: Large Language Models and the Future of Infectious Diseases Consultation. Clin Infect Dis Off Publ Infect Dis Soc Am 78(4):860–866. 10.1093/cid/ciad633 PMC11006107 37971399

[35] Timing of Antiretroviral Therapy after Diagnosis of Cryptococcal Meningitis | New England Journal of Medicine. Accessed October 7 (2025) https://www.nejm.org/doi/full/10.1056/NEJMoa1312884 10.1056/NEJMoa1312884 PMC4127879 24963568

[36] GBD 2021 Antimicrobial Resistance Collaborators (2024) Global burden of bacterial antimicrobial resistance 1990–2021: a systematic analysis with forecasts to 2050. Lancet Lond Engl 404(10459):1199–1226. 10.1016/S0140-6736(24)01867-1 PMC11718157 39299261

[37] Otaigbe II, Elikwu CJ (2023) Drivers of inappropriate antibiotic use in low- and middle-income countries. JAC-Antimicrob Resist 5(3):dlad062. 10.1093/jacamr/dlad062 37265987 PMC10230568

[38] Mulchandani R, Tiseo K, Nandi A (2025) Global trends in inappropriate use of antibiotics, 2000–2021: scoping review and prevalence estimates. BMJ Public Health 3(1):e002411. 10.1136/bmjph-2024-002411 40444029 PMC12121568

[39] Busch F, Hoffmann L, Rueger C (2025) Current applications and challenges in large language models for patient care: a systematic review. Commun Med 5(1):1–13. 10.1038/s43856-024-00717-2 39838160 PMC11751060

[40] Omar M, Sorin V, Collins JD (2025) Multi-model assurance analysis showing large language models are highly vulnerable to adversarial hallucination attacks during clinical decision support. Commun Med 5(1):330. 10.1038/s43856-025-01021-3 40753316 PMC12318031

[41] Kalai AT, Nachum O, Vempala SS, Zhang E (2025) Why Language Models Hallucinate. arXiv. Preprint posted online September 4. 10.48550/arXiv.2509.04664

[42] Generating credible referenced medical research: A comparative study of openAI’s GPT-4 and Google’s gemini - ScienceDirect. Accessed December 16 (2024) https://www.sciencedirect.com/science/article/pii/S0010482524016305 10.1016/j.compbiomed.2024.109545 39667055

[43] Gorenshtein A, Shihada K, Sorka M, Aran D, Shelly S (2025) LITERAS: Biomedical literature review and citation retrieval agents. Comput Biol Med 192:110363. 10.1016/j.compbiomed.2025.110363 40383055

[44] Gorenshtein A, Omar M, Glicksberg BS, Nadkarni GN, Klang E AI Agents in Clinical Medicine: A Systematic Review. medRxiv. Preprint posted online August 26, 2025:2025.08.22.25334232. 10.1101/2025.08.22.25334232

[45] Bloodstream Infections Published online 2025

[46] Urinary Tract Infection Published online 2025

[47] Siddig EE, Eltigani HF, Ahmed A (2023) The Rise of AI: How Artificial Intelligence is Revolutionizing Infectious Disease Control. Ann Biomed Eng 51(12):2636–2637. 10.1007/s10439-023-03280-4 37335374

[48] Omar M, Brin D, Glicksberg B, Klang E (2024) Utilizing natural language processing and large language models in the diagnosis and prediction of infectious diseases: A systematic review. Am J Infect Control 52(9):992–1001. 10.1016/j.ajic.2024.03.016 38588980

[49] Howard A, Reza N, Green PL (2025) Artificial intelligence and infectious diseases: tackling antimicrobial resistance, from personalised care to antibiotic discovery. Lancet Infect Dis 0(0). 10.1016/S1473-3099(25)00313-5 40972630

